# Sodium nitrate co-supplementation does not exacerbate low dose metronomic doxorubicin-induced cachexia in healthy mice

**DOI:** 10.1038/s41598-020-71974-z

**Published:** 2020-09-24

**Authors:** Dean G. Campelj, Danielle A. Debruin, Cara A. Timpani, Alan Hayes, Craig A. Goodman, Emma Rybalka

**Affiliations:** 1 Institute for Health and Sport, Victoria University, Melbourne, VIC Australia; 2 Australian Institute for Musculoskeletal Science (AIMSS), Victoria University, St Albans, Victoria, Australia; 3 Department of Medicine - Western Health, Melbourne Medical School, The University of Melbourne, Melbourne, VIC Australia; 4 Centre for Muscle Research (CMR), Department of Physiology, The University of Melbourne, Parkville, VIC Australia

**Keywords:** Physiology, Adverse effects, Chemotherapy, Combination drug therapy

## Abstract

The purpose of this study was to determine whether (1) sodium nitrate (SN) treatment progressed or alleviated doxorubicin (DOX)-induced cachexia and muscle wasting; and (2) if a more-clinically relevant low-dose metronomic (LDM) DOX treatment regimen compared to the high dosage bolus commonly used in animal research, was sufficient to induce cachexia in mice. Six-week old male Balb/C mice (n = 16) were treated with three intraperitoneal injections of either vehicle (0.9% NaCl; VEH) or DOX (4 mg/kg) over one week. To test the hypothesis that sodium nitrate treatment could protect against DOX-induced symptomology, a group of mice (n = 8) were treated with 1 mM NaNO_3_ in drinking water during DOX (4 mg/kg) treatment (DOX + SN). Body composition indices were assessed using echoMRI scanning, whilst physical and metabolic activity were assessed via indirect calorimetry, before and after the treatment regimen. Skeletal and cardiac muscles were excised to investigate histological and molecular parameters. LDM DOX treatment induced cachexia with significant impacts on both body and lean mass, and fatigue/malaise (i.e. it reduced voluntary wheel running and energy expenditure) that was associated with oxidative/nitrostative stress sufficient to induce the molecular cytotoxic stress regulator, nuclear factor erythroid-2-related factor 2 (NRF-2). SN co-treatment afforded no therapeutic potential, nor did it promote the wasting of lean tissue. Our data re-affirm a cardioprotective effect for SN against DOX-induced collagen deposition. In our mouse model, SN protected against LDM DOX-induced cardiac fibrosis but had no effect on cachexia at the conclusion of the regimen.

## Introduction

Anthracyclines are a class of chemotherapeutic agents that are currently utilized as treatments for multiple cancers due to their potent anti-cancer efficacy^1^. One of the most common anthracyclines in clinical use is doxorubicin (DOX), which inhibits topoisomerase-II, leading to DNA damage and cell cycle arrest^2^. DOX also promotes toxicity through the reduction of its quinone moiety to an unstable semiquinone, during its metabolism by NADH dehydrogenase/Complex I of the mitochondrial ETC. In this setting, it hijacks homeostatic redox cycling to induce formation of superoxide (O_2-_) anion radicals and subsequently increases production of reactive oxygen species (ROS) leading to oxidative damage^3^. Despite the robust anti-cancer effectivity of DOX, its staunch cytotoxic profile promotes non-specific, off-target effects in healthy tissue (in particular cardiac and skeletal muscle; for extensive reviews see^4,5^, respectively) limiting its clinical utility and increasing the risk of morbidity and mortality^6^. As such, there is warranted clinical interest in preserving cardiac and skeletal muscle health during DOX administration, and a variety of adjunct therapeutics have been purported to attenuate the negative side-effects of DOX on these tissues.

Of clinical interest, a potential therapeutic strategy to protect against DOX-induced cardiotoxicity is sodium nitrate (SN) co-supplementation. SN acts by augmenting the nitrate/nitrite/nitric oxide (NO) pathway via a nitric oxide synthase (NOS)-independent mechanism, to increase NO production^7,8^. Importantly, this strategy has been shown to have therapeutic cardioprotective effects in other pre-clinical models of cardiomyopathy^9–14^. Pertinent to DOX-induced cardiomyopathy, two foundational studies have been conducted exploring the cardioprotective potential of SN co-supplementation. Data has demonstrated benefits to left ventricular function secondary to promoting anti-oxidant activity, and reducing oxidative stress via the inhibition of lipid peroxidation and amelioration of mitochondrial Complex I dysfunction^15,16^. Despite the strong cardioprotective efficacy of SN co-supplementation against DOX-induced cardiotoxicity, it is currently unclear as to whether SN co-supplementation elicits protective effects against the skeletal muscle toxicity induced by DOX.

DOX-induced skeletal muscle toxicity is widely accepted to be a consequence of its metabolism at Complex I of the mitochondrial ETC, resulting in mitochondrial dysfunction secondary to the enhancement of oxidative stress. As a consequence, DOX treatment is associated with skeletal muscle atrophy/wasting, as well as functional deficits^17–25^ as characterized by reduced force production and increased susceptibility to skeletal muscle fatigue in pre-clinical animal models^26–29^. These data are consistent with clinical descriptions of exercise intolerance, a lesser capacity for activities of daily living and reduced quality of life in patients following chemotherapy treatment^30–32^. The functional benefits of SN co-supplementation on the skeletal muscular system in both rodents and humans is well documented, whereby enhanced NO bioavailability augments functional adaptations that lower the oxidative cost of exercise, subsequently improving fatigue resistance and exercise tolerance^33–35^. However, in skeletal myopathic conditions, SN co-supplementation has equivocally been shown to enhance myopathy^36,37^. In a pro-oxidant setting, akin to that induced by DOX administration, enhancing the bioavailability of NO alongside superfluous superoxide anions driven by DOX metabolism leads to peroxynitrite formation and the exacerbation of nitrostative stress^38^.Thus, an aim of this study was to determine whether SN co-supplementation could afford the same therapeutic benefit as that observed previously against DOX-induced cardiotoxicity, or whether SN co-supplementation would exacerbate myopathy as per our previous study in the *mdx* mouse model of Duchenne Muscular Dystrophy^37^.

A second aim of this study was to investigate the effects of low-dose, metronomic (LDM) DOX administration on skeletal and cardiac muscle. A current problem with pre-clinical research using rodent models to investigate DOX-induced toxicity surrounds the administration of a single maximum tolerable dose (MTD) injection of DOX to induce a severe scenario of toxicity and mortality^39^. This largely misrepresents the clinical scenario, in which DOX is administered repeatedly at a fractional dosage over a set time-course, to give a cumulative dose equivalent to the MTD. Recently, there has been interest in the LDM administration of chemotherapeutic agents to reduce systemic toxicity, whilst maintaining the anti-cancer efficacy of treatment^40,41^. Remarkably, there is few data derived from animal studies using LDM DOX administration to investigate potential therapeutic adjuncts against DOX-induced side-effects. As such, the clinical applicability of experimental adjuvants is largely unknown. In this study we utilized a murine model of LDM administration of DOX in an attempt to reduce the severe toxicity profile associated with the MTD model, whilst still being able to make insights into DOX-induced cardiac and skeletal muscle toxicity. Furthermore, utilizing a LDM model of DOX administration allows for a more translatable screening of potential therapeutic strategies to protect against the negative side-effects elicited to cardiac and skeletal muscle from DOX.

## Results

### Assessment of body composition indices, muscle and organ mass

**Figure 1:**
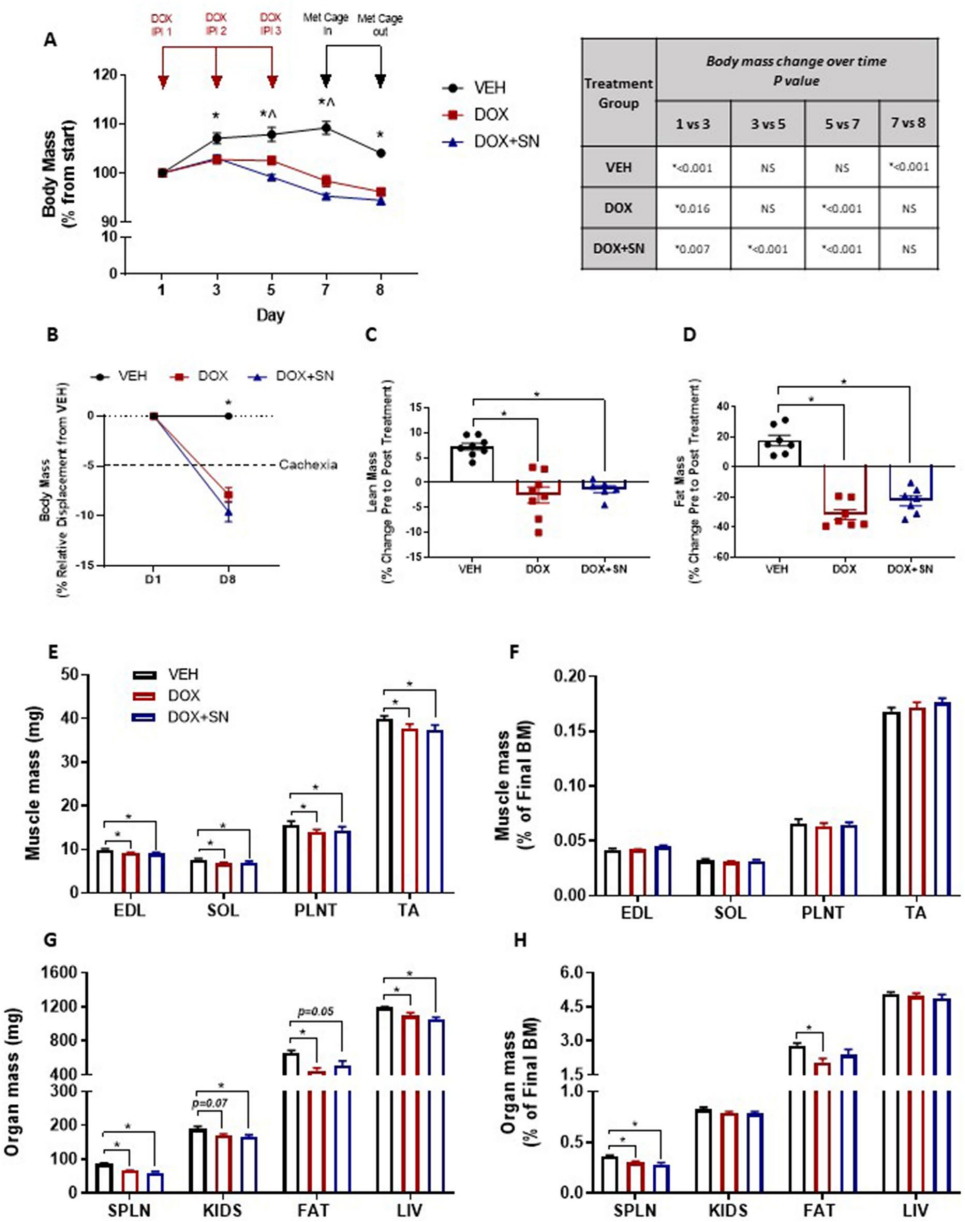
The effect of LDM DOX administration and SN co-supplementation on body mass and composition indices, muscle and organ mass*.* (**A**) Body mass was measured throughout the experimental timeline and repeated measures analysis performed. A 5% increase in body mass was observed between day 1 and 3 in the VEH mice (*p < *0.05*), which was inhibited by DOX such that by day 3, both DOX and DOX + SN mice had a lower body mass compared to VEH (*p < 0.05). Body mass was stable from day 3 to 7 in VEH mice, but declined steadily with DOX treatment from day 5 (*p < 0.05) and DOX + SN treatment from day 3 (*p < *0.05*). As such the body mass of DOX and DOX + SN mice was lower compared to VEH at day 5 and 7 (*p < 0.05). Weight loss during this period was actually exacerbated by SN supplementation (^*p* < 0.05 DOX + SN versus DOX). Body mass declined during the 24 h metabolic cage stay between day 7 and 8 in VEH mice (*p < *0.05*) due to participation in voluntary wheel running. Exercise-induced weight loss was not observed in DOX and DOX + SN mice (since they did not run as far as VEH mice) albeit body mass was still lower for these groups compared to VEH mice (*p < 0.05). (**B**) As defined by a > 5% reduction in body mass displacement relative to the VEH, LDM DOX and DOX + SN treatment induced cachexia (*^p < *0.05*). Mirroring body mass changes, both (**C**) lean and (**D**) fat mass was significantly lower in DOX and DOX + SN mice compared to VEH mice (*p < 0.05). (**E**) The mass of extensor digitorum longus (EDL), soleus (SOL), plantaris (PLNT) and tibialis anterior (TA) muscles was significantly reduced by DOX administered alone and in the DOX + SN treatment compared to VEH mice (*p < *0.05*). However, when (**F**) muscle mass was corrected for final body mass (BM), there was no treatment effect observed. Like muscles, organ mass was either lower, or trended to be lower following DOX treatment either alone or in the DOX + SN treatment for each of (**G**) spleen (SPLN; *p < *0.05* for DOX and DOX + SN compared to VEH), kidneys (KIDS; *p* = *0.07* DOX versus VEH and *p < *0.05* DOX + SN versus VEH), fat (epididymal and subcutaneous; FAT; *p < *0.05* for DOX versus VEH and *p* = *0.05* for DOX + SN versus VEH) and liver (LIV; *p < *0.05* for DOX and DOX + SN versus VEH). (**H**) When corrected for final BM, DOX treatment showed specific targeting of both SPLN (*p < *0.05* for both DOX and DOX + SN versus VEH) and FAT (*p < *0.05* for DOX versus VEH) which was independent of overall body mass reduction . *n* = 7–8 per group.

We found that LDM DOX administration induced growth inhibition initially (i.e. from day 1–5), which progressively led to a loss of body mass from day 5 to 7 (*p* < 0.05 DOX and DOX + SN different from VEH; Fig. 1A & Supp Fig. 1) that was exacerbated by SN supplementation (*p* < *0.05* DOX + SN from DOX; Fig. 1A and Supp Fig. 1). Whereas VEH mice lost body mass during the 24 h spent (i.e. from day 7 to 8) in the metabolic cages with access to running wheels, DOX and DOX + SN treated mice did not (Fig. 1A). By the experimental endpoint at day 8, DOX-treated mice were cachetic (*p* < 0.05; Fig. 1B), but cachexia was neither exacerbated nor alleviated by SN treatment (i.e. DOX and DOX + SN mice were comparable in the degree of body weight displacement from VEH mice; Fig. 1B). Unsurprisingly, DOX-induced cachexia was mirrored in body composition indices measured via PRE- and POST-treatment echoMRI scans. DOX administration induced significant lean and fat mass loss which could not be rescued by SN co-supplementation (*p* < *0.05*; Fig. 1C, D & Supp Fig. 1). Interestingly, while this wasting phenotype was characterised by both the loss of raw skeletal muscle mass (*p* < *0.01*; Fig. 1E) and organ mass (*p* < *0.0001*; Fig. 1G), skeletal muscle mass (Fig. 1F) was not a specific target of DOX like some organs were (Fig. 1H) with spleen and fat mass being the only tissues to waste disproportionately to body mass (*p* < *0.*05; Fig. 1H). There was no significant effect of SN co-supplementation on any measure of muscle or organ mass relative to DOX treatment alone (Fig. 1E–H). Our data highlight that LDM DOX administration induces a wasting phenotype that broadly impacts the skeletal muscles, adipose tissue and organs, but which specifically wastes the spleen and adipose tissue, which is neither rescued nor exacerbated by SN co-supplementation.

### Assessment of ambulatory and metabolic activity via indirect calorimetry

**Figure 2:**
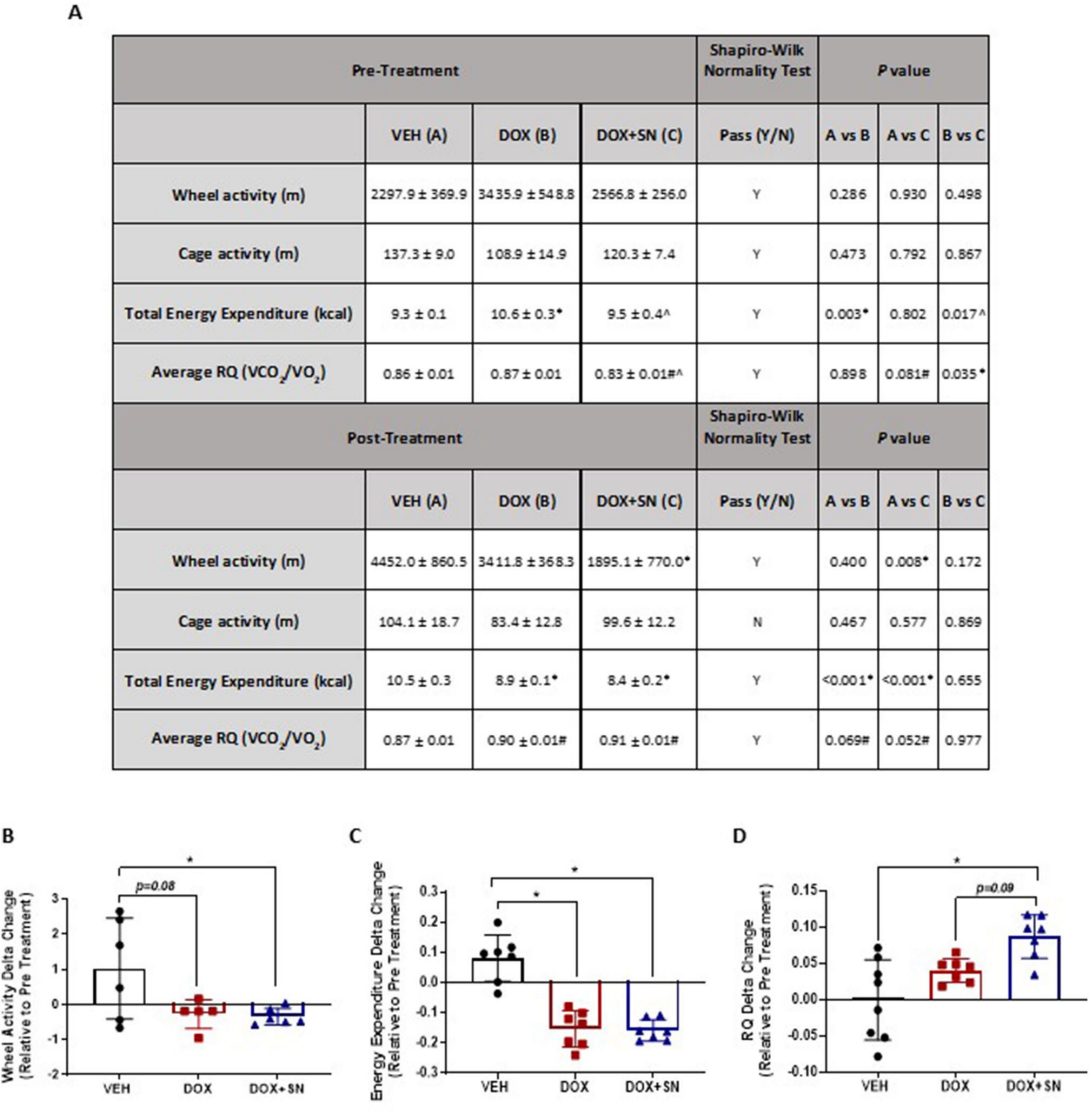
The effect of LDM DOX administration and SN co-supplementation on ambulatory and metabolic activity*.* (**A**) Data are displayed for ambulatory (wheel and cage meters) and metabolic activity (energy expenditure and respiratory quotient (RQ) parameters over 24 h in each of the PRE and POST treatment periods. (**B**) There was a strong trend for DOX to increase wheel running activity and a significant effect of DOX + SN treatment on wheel running activity in the PRE to POST treatment periods, (**C**) This was mirrored by significant reductions in energy expenditure between the PRE and POST treatment periods for both DOX and DOX + SN (*^p < *0.05*). (**D**) While there was no effect of DOX on the respiratory quotient (RQ), DOX + SN significantly increased the RQ in favour of glucose as an energy substrate compared to VEH (*p < *0.05*) and there was a strong trend for the same effect compared to DOX treatment alone (*p* = *0.09*); *n* = 5–7 per group.

DOX treatment has been previously shown to induce exercise intolerance in cancer patients^30^, while dietary nitrate supplementation is a potentiator of exercise performance in healthy humans^42^. Thus, in this study, we assessed the effects of LDM DOX and DOX + SN treatment on voluntary physical and metabolic activity over a 24 h period in mice. There was a strong trend for LDM DOX treatment to reduce wheel running activity between the PRE and POST periods compared to VEH (p = 0.08; Fig. 2A,B) and this effect was significant in the DOX + SN group (*p* < 0.05; Fig. 2A,B). The reduction in voluntary wheel running activity was concomitant with reduced energy expenditure in the PRE to POST treatment periods (*p* < *0.05*; Fig. 2A,C), which was interesting because the PRE-treatment energy expenditure of this particular cohort of mice was significantly higher than that of the VEH and DOX + SN groups (*p* < *0.05* for both; Fig. 2A). The DOX-induced reduction in energy expenditure was associated with reduced wheel-running activity (*p* < *0.05*; Fig. 2B) rather than changes to metabolism, as reflected by the RQ (Fig. 2D), and thus, is likely comparable to mental and physical fatigue reported by human patients during chemotherapy treatment^43^ rather than true physiological fatigue. SN co-supplementation had no effect on the DOX-induced changes in energy expenditure (Fig. 2A), but it did elevate the RQ compared to VEH (p,0.05; Fig. 2D) and there was a trend toward the same effect compared to DOX treatment alone (p = 0.09; Fig. 2D), suggestive of increased dependency on glucose as a substrate during activity.

### Assessment of cardiac muscle mass and histology

**Figure 3:**
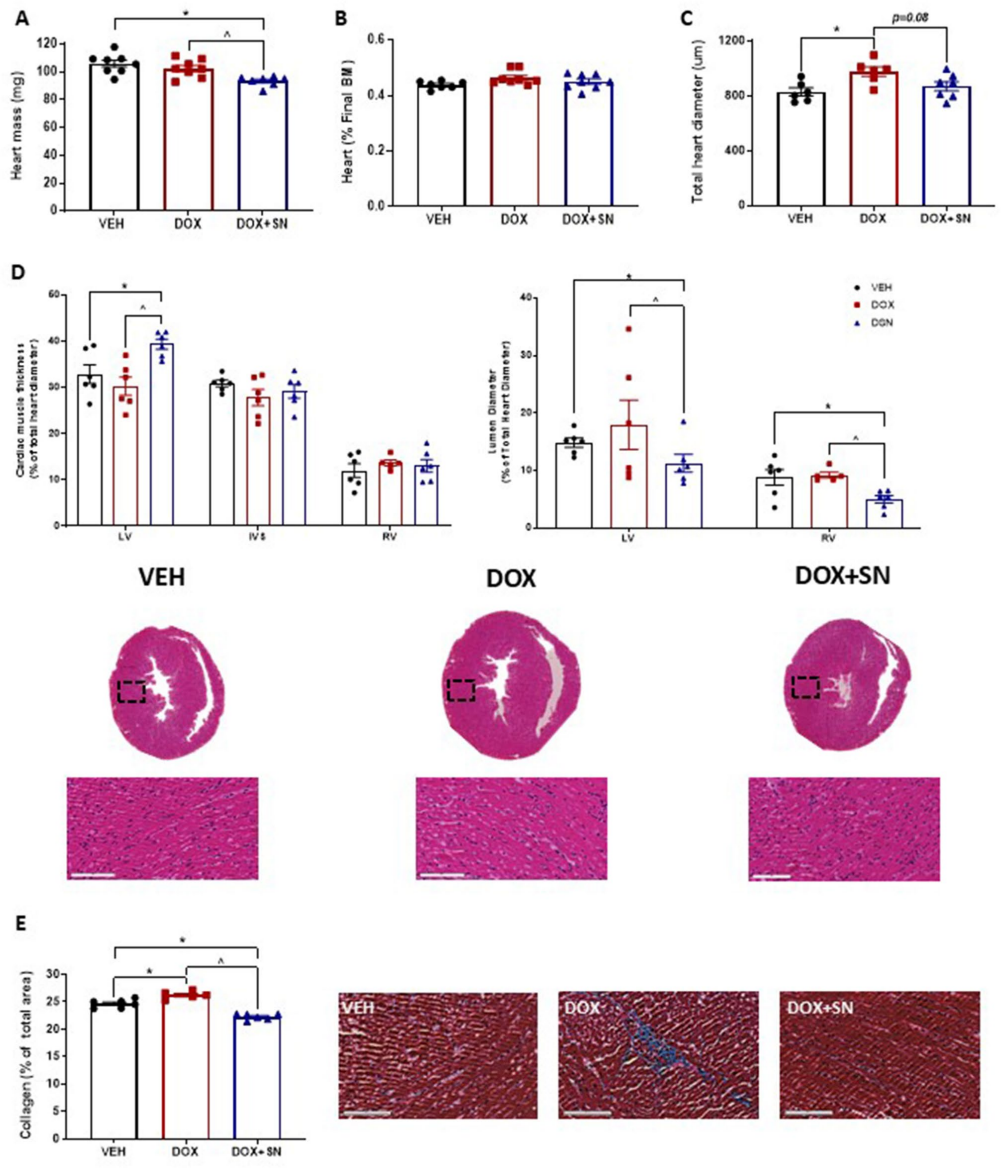
The effect of LDM DOX administration and SN co-supplementation on cardiac muscle histology. (**A**) Heart mass was weighed immediately post-harvest, with DOX + SN treatment reducing heart mass compared to VEH (*p < 0.05) and DOX (^*p* < 0.05) mice. (**B**) When expressed as a percentage of the final body mass (BM), there were no significant differences in heart mass between groups. Gross morphological cardiac muscle indices were evaluated from H&E stained sections, measured in triplicate and normalised to (**C**) total heart diameter, which was increased in DOX mice (*p < 0.05) compared to VEH. However, DOX + SN treatment had a tendency (p = 0.08) to correct this DOX-induced change back to VEH control levels. (**D**) Next, we made morphological measurements of the cardiac muscle and ventricular lumen components of the lower hear (expressed as a percentage of total heart diameter). DOX + SN treatment increased the wall thickness of left ventricle (LV) compared to VEH (*p < 0.05) and DOX (^*p* < 0.05) mice, however, the intraventricular septum (IVS) and right ventricular (RV) wall thickness were unchanged from treatments. DOX + SN treatment also reduced the diameter of the LV and RV lumen (*p < *0.05* DOX + SN versus VEH and *^p* < *0.05* DOX + SN versus DOX). (**E**) Representative images of the H&E sections displayed. (**F**) Heart sections were also stained using Masson’s trichrome to evaluate the relative percentage of collagen/fibrotic connective tissue. DOX-treated mice had a greater relative percentage of collagen (*p < 0.05) compared to VEH, while DOX + SN mice corrected this phenomenon by reducing the relative percentage of collagen compared to VEH (*p < 0.05) and DOX(^*p* < 0.05). *n* = 6–7.

Cardiotoxicity is a well-established side-effect of DOX treatment and severity is dose-dependent^44^. In our study, LDM DOX administration had no effect on heart mass (Fig. 3A,B), while SN co-supplementation reduced raw heart mass (*p* < 0.05, Fig. 3A), albeit this was not significant when normalised to body mass (Fig. 3B). We investigated histological changes induced by chemotherapy, with LDM DOX administration increasing the total diameter of the heart (*p* < 0.05; Fig. 3C). Interestingly, there were no significant effects elicited from LDM DOX administration on morphological cardiac muscle indices (Fig. 3D). LDM DOX administration did, however, increase collagen deposition in cardiac tissue (Fig. 3E), suggestive of early-stage myocardial fibrosis that would likely reduce the contractility of the heart^45,46^. SN has demonstrable therapeutic benefit in mitigating DOX-induced cardiotoxicity in mice when supplemented prior to, and following, the administration of a single MTD bolus of DOX^15^. In our study, DOX + SN treatment actually enhanced the DOX-induced reduction in heart mass (Fig. 3A), however, this appeared to be related to the larger body mass reduction observed with DOX + SN versus DOX-treatment alone since this effect was abolished when heart mass was normalised to body weight (Fig. 3B). We did observe a strong trend for DOX + SN treatment to mitigate the enlarged total heart diameter induced by DOX-treatment (p = 0.08; Fig. 3C), and this is likely related to the beneficial effect that SN co-supplementation had on collagen deposition through the cardiac tissue, in which collagen content was reduced compared to both DOX and VEH mice (*p* < 0.05; Fig. 3E). Interestingly, SN co-supplementation increased LV wall thickness compared to both VEH and DOX treatment (*p* < 0.05; Fig. 3D), however it significantly reduced the LV and RV lumen diameter (*p* < *0.05* DOX + SN versus VEH and DOX; Fig. 3D). Overall, our data suggest that SN co-supplementation has protective effects against a mild pro-collagenic driven cardiofibrosis induced by LDM DOX treatment, which may be related to the smaller heart size.

### Assessment of skeletal muscle histology

**Figure 4:**
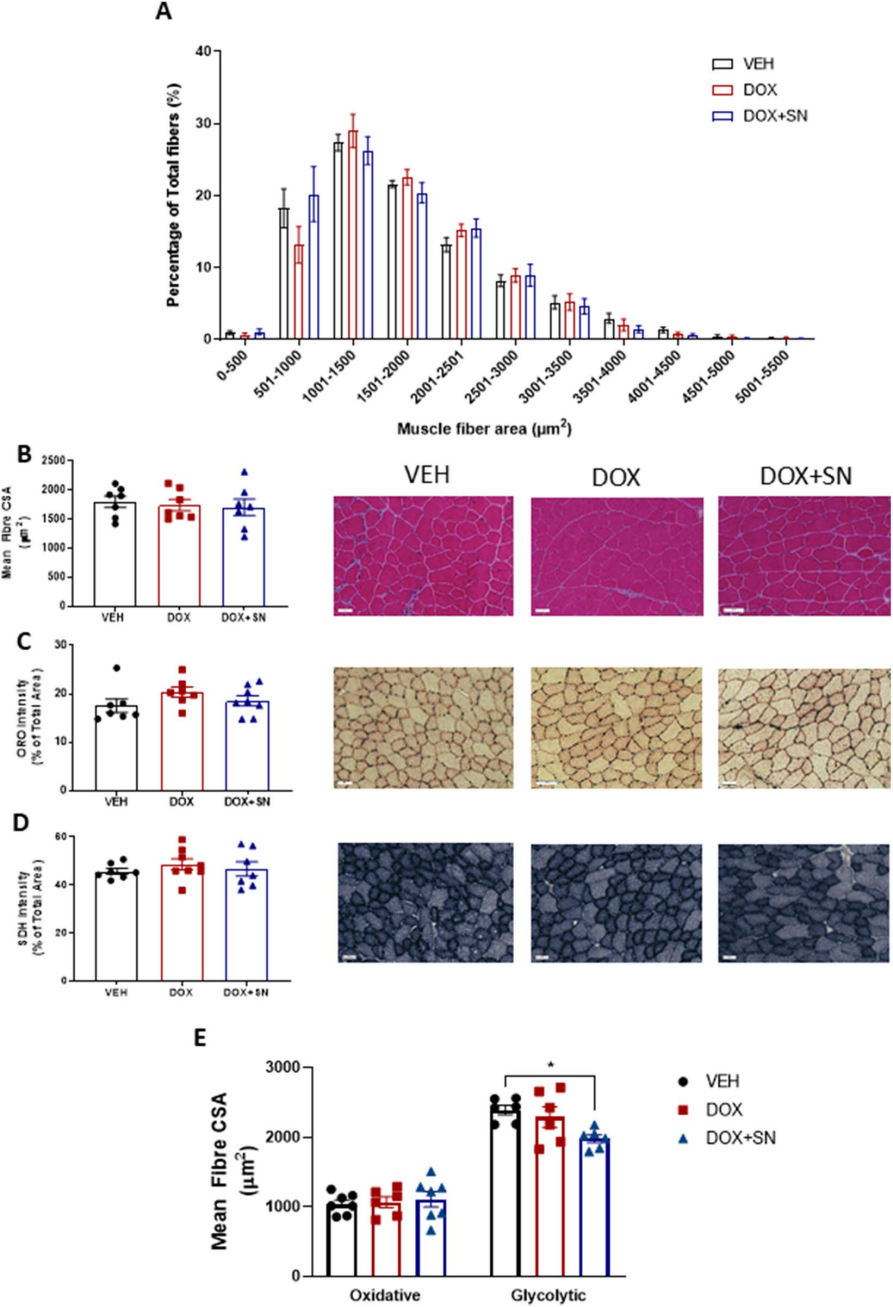
The effect of LDM DOX administration and SN co-supplementation on skeletal muscle histology*.* Tibialis anterior (TA) muscles were cryosectioned and underwent H&E staining to assess skeletal muscle architecture and fibre cross-sectional area (CSA). (**A**) The relative distribution of fibre size normalized as a percentage of total fibres; and (**B**) mean muscle fibre cross sectional area (CSA); demonstrated no significant differences between groups. Skeletal muscle cross sections also underwent (**C**) Oil Red O (ORO) staining to evaluate lipid content; and (**D**) Succinate Dehydrogenase (SDH) staining to evaluate mitochondrial content. There were no significant effects of treatments on these indices. (**E**) To assess whether fibre types were affected differently by either DOX alone of with SN supplementation, SDH-stained sections were used to determine the CSA of isolated oxidative and glycolytic fibre populations. There was no effect of DOX treatment on the CSA of either oxidative or glycolytic fibres, however, DOX + SN treatment decreased the mean CSA of glycolytic fibres (*p < *0.05*) *n* = 6–8.

Neither DOX administration, nor SN co-supplementation, had a significant effect on the fibre CSA or skeletal muscle architecture of the TA when stained with H&E (Fig. 4A & B). Furthermore, there was no change in fat deposition, measured via ORO staining (Fig. 4C), nor mitochondrial abundance/activity, as indicated by SDH staining (Fig. 4D) from DOX administration or SN co-supplementation. To determine whether there were differential effects of DOX with and without SN co-supplementation on oxidative versus glycolytic fibres, we conducted CSA analysis on distinct populations of these fibre types based upon SDH staining intensity. There was no effect of DOX on the CSA of either oxidative or glycolytic fibres (Fig. 4E). DOX + SN treatment significantly reduced the CSA of glycolytic fibres (*p* < *0.05* DOX + SN versus VEH; *p* = *0.07* DOX + SN versus DOX; Fig. 4E) but had no effect on oxidative fibres, suggestive of nitrate-mediated fibre type transformation from IIb to IIa type.

### Assessment of molecular redox signalling pathways in skeletal muscle

**Figure 5:**
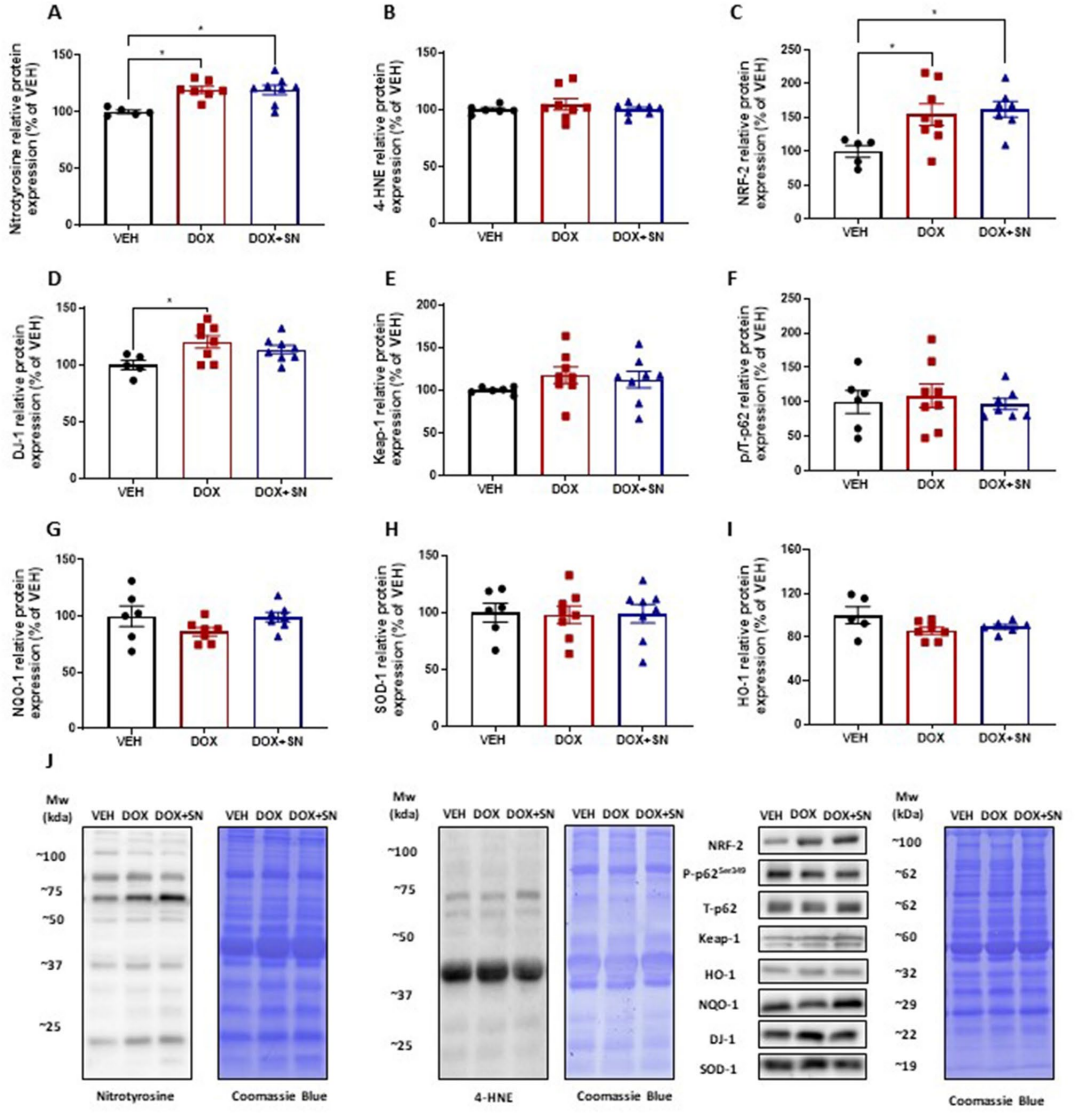
The effect of LDM DOX administration and SN co-supplementation on molecular markers of oxidative stress and the anti-oxidant response in skeletal muscle. Western blotting experiments were undertaken utilizing soleus muscle homogenate. When probing for molecular markers of oxidative stress, there was no significant change in (**A**) 4-HNE protein expression, a marker of lipid peroxidation, between treatment groups, but there was a significant increase in (**B**) nitrotyrosine protein expression, a marker of excessive peroxynitrite (ONOO^-^) production in DOX and DOX + SN treated mice (*p < 0.05) compared to VEH. There was also a significant increase in (**C**) NRF-2 protein expression, the master regulator of the anti-oxidant response to cellular stress, in DOX and DOX + SN treated mice (*p < 0.05) compared to VEH. (**D**) DJ-1 protein expression was probed for as a positive regulator of NRF-2 activation, which was interestingly, shown to increase in DOX mice (*p < 0.05) compared to VEH, whilst there was no significant change in DOX + SN treated mice. Negative regulators of NRF-2, i.e. (**E**) Keap-1, (**F**) phosphorylated p62^ser349^ and total p62 (depicted as a ratio of the total), were not significantly changed from treatment. Downstream targets of the anti-oxidant response, i.e. (**G**) NQO-1, (**H**) SOD-1 and (**I**) HO-1 did not significantly change from treatment. (**J**) Representative images of the antibodies are displayed alongside a representative image of Coomassie Blue used to normalize to total protein content. *n* = 5–8.

Metabolised by Complex I of the mitochondrial ETC, DOX is a well-known inducer of superoxide production as a by-product of its metabolism^4,5,13^. Similarly, we have previously demonstrated that in a pro-oxidant environment, SN co-supplementation has a damaging effect on skeletal muscle secondary to a higher peroxynitrite production^37^. Thus, we next investigated whether oxidative or nitrostative stress played a role in the DOX-induced reduction in energy expenditure. Interestingly, there was no effect of LDM DOX administration on 4-hydroxy-2-nonenal (4-HNE; Fig. 5A,J), a marker of lipid peroxidation. However, there was a significant increase in nitrotyrosine (*p* < *0.*05; Fig. 5B,J), a marker of excessive peroxynitrite (ONOO^-^) production. Surprisingly, SN co-supplementation did not augment the DOX-induced increase in nitrotyrosine expression (*p* < *0.*05; Fig. 5B,J). LDM DOX administration also significantly increased the expression of the redox status sensor, nuclear factor erythroid-2-related factor 2 (NRF-2; *p* < *0.*05; Fig. 5C,J), and this was unchanged by SN co-supplementation. Since NRF-2 is the master regulator of the anti-oxidant response to cellular stress^47^, we wanted to investigate changes to regulators or downstream targets of NRF-2. The protein expression of the negative regulator of NRF-2, Keap-1 (Fig. 5D,J), nor the serine 349 residue on p62 (Fig. 5E,J), which plays a role in balancing the NRF-2 and Keap-1 interaction, did not alter in response to LDM DOX administration. Interestingly, we were able to demonstrate that LDM DOX administration significantly upregulated DJ-1 protein expression, which is a positive regulator of NRF-2 activation through enhancing NRF-2 stability and reducing ubiquitination and Keap-1 dependent degradation^48,49^, but not when co-supplemented with SN (*p* < *0.*05; Fig. 5F,J). The protein expression of anti-oxidant enzymes that are transcriptional targets of NRF-2, being NAD(P)H dehydrogenase quinone-1 (NQO-1), superoxide dismutase-1 (SOD-1) and heme oxygenase-1 (HO-1) (Fig. 5G-J), were not altered by LDM DOX administration nor SN co-supplementation.

### Assessment of molecular mitochondrial content signalling in skeletal muscle

**Figure 6:**
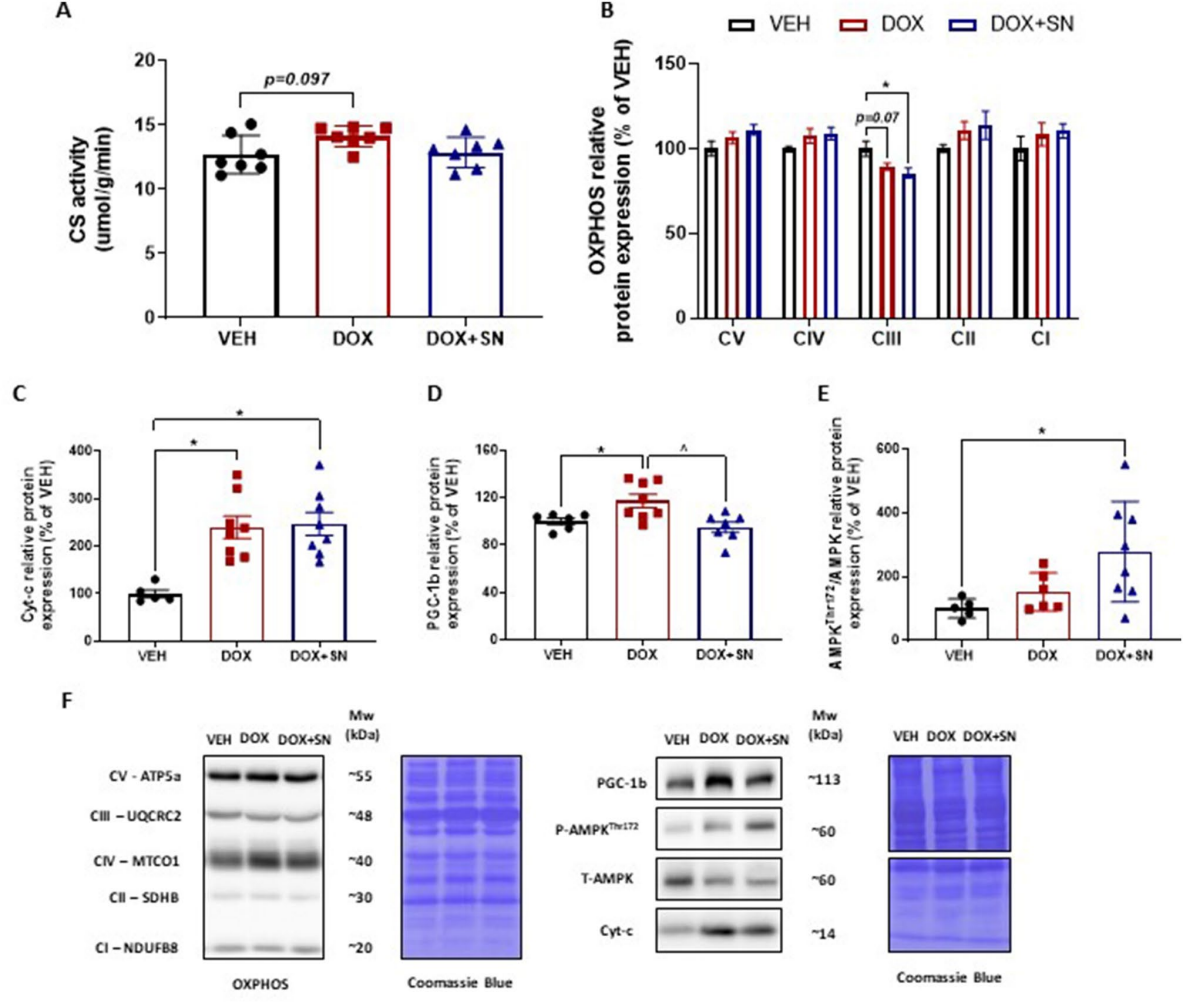
The effect of LDM DOX administration and SN co-supplementation on molecular markers of mitochondrial content in skeletal muscle*.* Western blotting experiments were undertaken utilizing soleus muscle homogenate. (**A**) CS activity was assessed as a gold standard marker of mitochondrial content. There was a trend towards increased CS activity induced by DOX (p = 0.07) but no effect of DOX + SN. Molecular indicators of mitochondrial complex protein content were probed for using the (**B**) OXPHOS cocktail, with no significant change in the protein expression of Complex V (CV)—ATP5A, Complex IV (CIV)—MTCO1, Complex II (CII)—SDHB and Complex I (CI)—NDUFB8 from treatment groups. However, the protein expression of Complex III (CIII)—UQCRC2 displayed a trend to be reduced in DOX mice (*p* = 0.07) compared to VEH, but was significantly reduced in DOX + SN treated mice (*p < 0.05) compared to VEH. Additionally, (**C**) Cytochrome-c (Cyt-c) protein expression, a marker of mitochondrial content, was significantly increased in both DOX and DOX + SN mice (*p < 0.05) compared to VEH. To assess mitochondrial remodelling, we also probed for a member of the PGC-1 family, i.e. (**D**) PGC-1β, which was shown to be significantly increased in DOX treated mice (*p < 0.05), but was normalised in DOX + SN mice compared to DOX (^*p* < 0.05) and VEH (p > 0.05). As a marker of metabolic stress, we also probed for (**E**) phosphorylated (i.e. activated) compared to total adenosine monophosphate activated protein kinase (AMPK) and showed no effect of DOX treatment alone, but a marked increase with DOX + SN treatment compared to VEH (*p* < 0.05). (**F**) Representative images of the antibodies are displayed alongside a representative image of Coomassie Blue used to normalize to total protein content. *n* = 5–8.

In light of the observation that administration of the LDM DOX regimen increased NRF-2 protein expression in skeletal muscle of mice with and without SN co-supplementation, and NRF-2 can modulate mitochondrial function and maintenance, we wanted to investigate whether molecular markers of mitochondrial content and remodelling were also affected. While we saw a trend for DOX to increase citrate synthase (CS) activity (*p* = *0.097*; Fig. 6A), the gold standard marker of mitochondrial content^50^ , there was no change to the protein expression of Complex I, II, IV and V from treatment groups (Fig. 6B, F) suggesting unchanged mitochondrial content. However, there was a strong trend for LDM DOX administration (*p* = 0.07; Fig. 6B) to reduce Complex III protein expression, whilst DOX + SN treatment did significantly reduce Complex III protein expression (*p* < 0.05; Fig. 6B, F). To assess damage to the mitochondrial pool, we also probed for cytochrome C (Cyt-c), which was significantly increased by both LDM DOX and DOX + SN treatment (*p* < 0.05, Fig. 6C, F). Following on from this finding we wanted to investigate if there were any effects on molecular markers of mitochondrial remodelling and stress, hence we probed for a member of the PGC-1 family (PGC-1β) and activation (phosphorylation) of adenosine monophosphate-activated protein kinase (AMPK). PGC-1β was significantly increased from LDM DOX administration (*p* < *0.05*, Fig. 6D,F) but was normalised to VEH control levels by SN co-supplementation (*p* < 0.05, Fig. 6D,F). AMPK activation was unaffected by DOX treatment but was significantly increased by SN supplementation (*p* < *0.05*; Fig. 6E,F).

## Discussion

The major findings in this study are that the LDM DOX chemotherapy regimen is sufficient to reduce body, lean and fat mass (i.e. induce cachexia) and cause malaise, as exhibited by reduced energy expenditure and wheel running activity. This study also supports the two main studies in the literature advocating the efficacy of SN co-supplementation as a therapeutic strategy to mitigate DOX-induced cardiofibrosis^15,16^. SN co-supplementation protected against a LDM DOX-induced collagen deposition within the heart, whilst not exacerbating the reduction in end-point body composition indices and muscle/organ mass. SN co-supplementation did not produce a beneficial effect, nor was it detrimental at the skeletal muscle level and it was unable to attenuate DOX-induced cachexia.

In this study, we were able to induce a progressive form of chemotherapy–induced cachexia with LDM DOX administration, as defined by the > 5% reduction in end-point body mass which was driven by reductions in both lean and fat mass^51^ and was unaffected by SN co-supplementation. The wasting of lean tissue by LDM DOX administration was predominately driven by visceral organ (spleen, liver, kidney) mass loss, with no impact on hindlimb skeletal muscle mass relative to body weight, albeit hindlimb muscle mass was reduced by DOX commensurate with the loss of body mass. We saw no evidence of muscle fibre atrophy (i.e. fibre CSA) of TA muscles. While this finding was unexpected, we postulate that skeletal muscle toxicity (similar to cardiac muscle toxicity) is dose dependent^44^ since other studies that utilized a higher cumulative dose of DOX (i.e. 20–30 mg/kg) have demonstrated significant skeletal muscle wasting^17–20^. Despite the lack of evidence for skeletal muscle wasting/atrophy in this model, it is still of interest that our model of LDM DOX administration (12 mg/kg; total cumulative dose over 7 days) can have a substantial acute effect on body and lean (visceral) mass i.e. an ~ 9% reduction compared to VEH control. This is comparative to a study by Nissinen et al. which utilized a cumulative dose of 24 mg/kg over 4 weeks and demonstrated similar body and lean mass loss, although evidence of skeletal muscle mass loss was also observed^18^ suggesting that skeletal muscle wasting induced from DOX administration is both a time and dose dependent event. Supporting this statement is a recent study by Tarpey et al.^29^, which utilized a single MTD injection of DOX (20 mg/kg) with mice culled 72-h post-injection, to demonstrate a significant reduction in body mass but no change in the mass or CSA of hindlimb skeletal muscle. Thus, the LDM DOX regimen utilised in this study represents a more clinically-relevant rodent model of DOX-induced cachexia in being able to induce similar body composition changes, without the severe systemic toxicity associated with the single MTD injection model^39^. Further improvement to the LDM DOX administration model could involve increasing the cumulative dose while administering DOX over both a longer duration, and for multiple LDM cycles, to enhance the potential for the effective pre-clinical screening of therapeutics to mitigate the side-effects of chronic DOX-induced toxicity.

The regimen of LDM DOX administration used in our study allowed the observation of an acute mild pro-collagenic cardiomyopathy involving early changes to the overall heart diameter, but with no change to crude heart mass. SN co-supplementation elicited a protective effect by preventing the induction of myocardial fibrosis. The increase in fibrotic tissue prevalence in the heart is a consistent hallmark of DOX-induced cardiotoxicity^52–56^, and our data demonstrate this precedes DOX-induced myocardial toxicity. Surprisingly, SN co-supplementation increased left ventricular wall thickness compared to both the VEH and DOX control group. We postulate that this finding is relative to enhanced left ventricular performance, which has previously been demonstrated by Zhu et al., where SN supplemented prior to, and following, the administration of a single MTD bolus of DOX, improved left-ventricular ejection fraction and fractional shortening through improved Ca^2+^ cycling and contractility^15^. Interestingly, SN co-supplementation reduced total heart diameter, heart mass and the left and right ventricular lumen diameter relative to the DOX control group, which we hypothesise is part of the potential protective effect of SN co-supplementation during LDM DOX administration. In this regard, maintaining a smaller, but more efficient heart may be beneficial for resisting the cardiac fibrosis induced by DOX treatment. Indeed, smaller stature humans and animals are thought to be more resistant to cardiovascular disease than their taller/larger counterparts^57^.

Fatigue and malaise represents a complex interplay between central nervous system drive, mental state and physiological function, and with chemotherapy treatment, is further complicated by nausea and a general feeling of unwell alongside altered gut function, which impacts macro and micro nutrient intake and uptake^58–60^. Chemotherapy administration induces chronic and debilitating fatigue, and this manifests physiologically in the skeletal muscular system as contractile and mitochondrial dysfunction (reviewed by us previously^25^). Thus, “fatigue” in the context of the present study likely represents the inability to generate both the mental and cellular energy to perform normal skeletal muscle function rather than true physiological fatigue, whereby normal skeletal muscle function cannot be durably maintained despite maximal effort. In this study, LDM DOX administration reduced voluntary wheel activity and energy expenditure in a synergistic manner, which was not alleviated by SN co-supplementation. DOX has previously been shown to impair skeletal muscle function and wheel running performance in mice, and this has been associated with mitochondrial dysfunction resulting from changes to the redox status of skeletal muscle^17,20,29,61,62^. In our study, DOX-induced “fatigue” was associated with a molecular marker of increased nitrosative stress (i.e. abundance of proteins with nitrosylated tyrosines), an indicator of increased peroxynitrite (ONOO^-^) levels. Excess peroxynitrite production can lead to impaired mitochondrial energy metabolism via inhibition of glycolysis and depletion of ATP pools^63^. Interestingly, SN co-supplementation did not further exacerbate the DOX-induced increase in nitrotyrosine expression, which was surprising given that we have previously demonstrated an escalation of muscle damage when SN was supplemented to an already pro-oxidant environment in the dystrophic *mdx* mouse model of Duchenne Muscular Dystrophy^37^. The postulated divergence in efficacy of SN co-supplementation between this study and our previous work in the *mdx* mouse is two-fold; with the first being acute versus chronic co-supplementation (i.e. 1 versus 8 weeks in this versus our *mdx* study) which may be protective rather than promotive of oxidative/nitrosative stress. The second factor is that the dynamics of the pro-oxidant environment may be influential. Dystrophic muscle is in a chronic state of oxidative stress, thus superfluous nitrate promoted excess peroxynitrite formation which exceeded the antioxidant buffering capacity, subsequently escalating myopathy. In contrast, LDM DOX administration which was metronomic and of reasonably short duration allowed for redox balancing in between doses in this study.

Surprisingly, the LDM regimen of DOX administration utilized in this study did not induce the expected increase in lipid peroxidation (a marker of oxidative stress), as indicated by 4-HNE protein expression. There have been mixed results in the literature to this effect, with Smuder et al. displaying a striking increase in 4-HNE protein expression when mice were culled 24 h post a single 20 mg/kg injection of DOX^23^, whereas Gouspillou et al. were unable to show changes to 4-HNE protein expression when mice were treated with a cumulative dose of DOX, i.e. 40 mg/kg over 12 weeks^20^. Collectively, these data in conjunction with ours suggest that LDM DOX delivery induces hormesis through NRF-2 to protect the muscle from oxidative stress, and that DJ-1, which has antioxidant properties^48,49^, is central to the hormetic response at the low DOX dose given in our study. In contrast, cytotoxic insults (i.e. single bolus MTD DOX treatment) overwhelms endogenous cytoprotective mechanisms to induce Phase II antioxidant enzymes such as SOD^23^.

We have previously demonstrated that DOX induces mitochondrial toxicity and reduces the mitochondrial pool in cultured myoblasts and myotubes^64^. In this study, we assessed the mitochondrial content by probing CS activity and mitochondrial ETC complex density in SOL and SDH content in TA, mitochondrial toxicity by probing Cyt-c, as well as the mitochondrial remodelling marker, PGC1β. We saw no change from either LDM DOX administration, nor SN co-supplementation, in the mitochondrial content of slow type I SOL muscle or the SDH content of whole fast type II TA cross-sections. However, both Cyt-c and PGC1β were elevated by DOX treatment highlighting the onset of mitochondrial toxicity and turnover as shown previously following the administration of various chemotherapeutic agents to human breast cancer cell lines^65^. Sanchez-Alcazar et al. have shown that increased expression of Cyt-c within the mitochondria and induction of PGC1-mediated mitochondrial biogenesis is an early cytoprotective response to anticancer agents, preceding Cyt-C release into the cytoplasm and the induction of apoptosis^65^. LDM DOX administration appears sufficient to induce mitochondrial toxicity but also turnover to preserve skeletal muscle mass.

Surprisingly, and in contrast to the expression profile of all other mitochondrial ETC complex subunits, we observed a reduction in the protein expression of Complex III following DOX treatment (with and without SN). The reduction in the Complex III subunit, UQCRC2, was an unexpected finding, as previous studies have shown that after a single MTD injection of DOX neither the protein expression of UQCRC2 in permeabilized myofibers^62^, nor the native protein content of Complex III in isolated mitochondria from skeletal muscle, is altered^29^. However, we postulate that the reduction in UQCRC2 content from LDM administration and SN co-supplementation could be a molecular marker of the exercise intolerance shown by DOX-treated mice in our activity/calorimetry studies. Others have shown increased UQCRC2 expression in the skeletal muscle of young mice exposed to cages with voluntary running wheels^66^ and that Complex III activity is reduced in sedentary mice compared to mice that have undergone treadmill running-based exercise training^67^. Interestingly, humans with mutations on the cytochrome b subunit of the Complex III assembly, display exercise intolerance and have a reduced protein expression of the UQCRC2 subunit in skeletal muscle^68–71^.

NO signalling in skeletal muscle is strongly associated with glucose uptake and utilisation during contraction^72^ which may be beneficial in circumstances such as during DOX administration, where mitochondrial dysfunction, damage, toxicity and ROS production escalate and energy production capacity is compromised. We have previously demonstrated that nitrate supplementation can augment contraction-induced glucose uptake in healthy type II skeletal muscles, but rather has a suppressive effect on glucose uptake in the pro-oxidative environment of dystrophic *mdx* type II skeletal muscles^37^. While we have not undertaken glucose uptake or contraction studies here, the activation (phosphorylation) of AMPK in DOX + SN treated SOL along with the higher RQ derived from calorimetry studies during voluntary wheel running activity, suggest a greater dependency on glucose metabolism following SN supplementation. Consistent with the known effects of nitrate supplementation on muscle fibre type switching from fast glycolytic (i.e. type IIb) to fast oxidative glycolytic (type IIa) phenotype^73^, we have also demonstrated a reduction in the CSA of glycolytic fibres. These adaptations could be of benefit to skeletal muscles long term, particularly in mitigating the contractile dysfunction and higher fatiguability reported by others in mice^17,27^ and humans^74^ following chemotherapy administration.

There were several limitations of our research worthy of mention. Firstly, our study examined the effects of LDM DOX administration on skeletal and cardiac muscle in an attempt to generate more clinically-relevant data than traditional single bolus MTD DOX administration which is typically given in animal studies to elicit skeletal muscle wasting and cardiotoxicity: in hindsight, our study would have benefited greatly from including a MTD group treated both with and without SN to contrast the DOX delivery regimens and the efficacy of SN against them. Secondly, our choice of limb muscles with different fibre-type profiles makes it difficult to compare and contrast our histological data (derived from the predominantly fast type II fibre TA muscle) with our molecular data (derived from the predominantly slow type I SOL muscle). TA is a common choice for histological analyses in mouse hind limb muscle because it is of sufficient size to rigorously generate fibre size distributions (i.e. to count enough (> 250) fibres). We chose SOL for our molecular studies since these centred around mitochondrial and oxidation markers which are more pronounced in slow oxidative muscle—we have undertaken CSA analyses on oxidative (slow type I) and glycolytic (fast type II) fibre populations and demonstrated no differential effects of DOX on specific fibre types though. Thirdly, we used mice aged ~ 6w of age which is prior to complete sexual maturation at 8w of age. Therefore, the effects observed on body, lean and fat mass indices may be different at this age when mice are still growing, versus in mature adult or aging mice when growth has ceased.

In conclusion, we demonstrate that the LDM DOX chemotherapy regimen is sufficient to induce cachexia (lean and fat mass wasting inclusive) and fatigue as characterised by reduced participation in voluntary exercise, which appears to be underlined by oxidative/nitrostative stress. Interestingly, the administration of LDM DOX induced mild oxidative stress in skeletal muscle, which subsequently evoked a NRF-2 driven anti-oxidant response through DJ-1. SN co-supplementation afforded no protective therapeutic potential alongside the administration of LDM DOX, but nor did it promote the wasting of lean tissue. Importantly, we have evidence of a SN co-supplementation driven cardioprotective effect against DOX-induced collagen deposition, however, the therapeutic efficacy of SN as an adjunct during DOX administration still requires further examination.

## Methods

### Animals

#### Experimental design and treatments

Six-week old male Balb/c mice were acquired from the Animal Resource Centre (ARC, Western Australia) and were randomly allocated to treatment groups (n = 8) upon arrival. Mice were housed on a 12-h light/dark cycle with ad libitum access to food i.e. standard mice chow and water supply throughout the experiments. Mice were administered with either VEH (0.9% NaCl) or DOX (4 mg/kg in 0.9% NaCl; Sigma Aldrich, Australia) via intraperitoneal injection 3 times over a 7 day period (i.e. on day 1, 3 and 5; for a cumulative dose of 12 mg/kg) which is the equivalent to a low cumulative clinical dose^5,75^, thus depicting a model of LDM DOX administration. SN was co-supplemented in drinking water ( 85 mg/L^−1^ (1 mM) (Sigma Aldrich, Australia)) throughout the duration of the LDM DOX regimen in a third group of animals (DOX + SN group). This dose of SN is equivalent to that used previously by us and others, demonstrating efficacy to transiently increase plasma and skeletal muscle nitrate levels^37,76^. Animals were weighed prior to the commencement of treatment (PRE), on each day of treatment and at the experimental endpoint. Food and water consumption were monitored throughout the duration of the treatment protocol.

### Body composition analyses

Echo Magnetic Resonance Imaging (echoMRI) was utilized to assess the effect of DOX and DOX + SN on body composition. Live mice were placed into an echoMRI body composition analyzer (EMR-150, Echo Medical Systems, USA) on day 1 (PRE) and day 8 (POST) of the treatment protocol. Total lean and fat mass were quantified via triplicate scans spaced 30 s apart. Data are the delta change in the mean of triplicate scans between the PRE and POST testing periods.

### Indirect calorimetry & activity monitoring

To evaluate the impact of DOX and DOX + DOX + SN on the physical and associated metabolic activity of mice, animals were individually housed for 24 h in Promethion Metabolic cages (Sable Systems, LV, USA) on day 0–1 (PRE) and day 7–8 (POST). Cages allowed free access to food, water and a running wheel. Real-time voluntary activity and whole-body metabolic activity was monitored throughout both 24-h periods, with the key variables of wheel running, ambulatory activity (pedestrian meters), energy expenditure and the respiratory quotient (VCO_2_/VO_2_) analysed as described by us previously^77^. Data presented are the total for a circadian cycle (i.e. of the diurnal phase recorded from 7am-7 pm and the nocturnal phase recorded from 7 pm-7am) in the PRE and POST periods, or the delta change between the PRE and POST periods..

### Surgery

At the conclusion of treatment (POST) live analyses, non-recovery surgery was performed on animals. Mice were deeply anaesthetised via isoflurane inhalation (5% induction and 2–3% maintenance) and non-survival surgery was performed. Tissues of interest were surgically excised, weighed and snap-frozen for post-hoc analyses in the following order: (1) SOL muscle was taken for western blotting experiments; and (2) TA muscle and heart were taken for histological assessment. Additional tissues of interest harvested were skeletal muscles *extensor digitorum longus* (EDL) and *plantaris* (PLNT), alongside epididymal and subcutaneous fat, spleen, kidney and liver, which were also weighed prior to being snap-frozen.

### Histological analyses

#### Skeletal muscle histology

All histological protocols were performed as described by us previously^37,78^. To determine whether LDM DOX had atrophic effects on skeletal muscle and subsequently, whether DOX + SN either exacerbated or rescued any such atrophy, we next assessed the hindlimb muscle, TA histologically. TA muscles were cryopreserved in optimal cutting temperature compound (Sakura Finetek) using liquid nitrogen-cooled isopentane. TA’s were sectioned (10 µm, -20 °C, Leica CM1950) and mounted. Three histological stains were employed to assess various histopathological features. Haematoxylin & Eosin (H&E) was utilised to evaluate muscle fibre size and architecture. Oil Red O (ORO) staining evaluated lipid content within the whole muscle. Succinate dehydrogenase (SDH) staining was utilized to assess the SDH activity, which is indicative of a more oxidative phenotype and subsequently a marker of both mitochondrial density and fibre-type transformation. For H&E, ORO and SDH, slides were imaged on a Zeiss Axio Imager Z2 microscope (Carl Zeiss MicroImaging GmbH, Germany) at 20 × magnification, respectively. All images were analysed using ImageJ software (NIH, USA).

To evaluate fibre type-specific effects of DOX and DOX + SN treatment, glycolytic and oxidative areas of the TA were identified in SDH stained sections and at least 250 fibres were counted for each fibre type (i.e. a total of at least 500 fibres per section) in each of those areas. The CSA of these fibres was determined as described by us previously^37,78^.

### Cardiac muscle histology

The cardiotoxic effects of DOX are well established, and previous research suggests a therapeutic effect for SN in this regard. Thus, in this study, we also sought to investigate the impact of our treatments on the heart. Whole hearts were snap-frozen before being transferred to a 10% neutral buffered formalin for 48 h for fixation. Once fixed, serial sections were cut at 10 µm diagonally at the mid-ventricle. H&E staining was performed to assess gross changes in cardiac muscle, with morphological indices measured including left and right ventricular diameter and thickness as well as interventricular septum thickness^79^. Masson’s Trichrome staining was performed to assess collagen/fibrotic connective tissue content. All sections were imaged at × 20 using a Zeiss Axio Imager Z2 microscope (Carl Zeiss MicroImaging GmbH, Germany). Masson’s trichrome stained sections were quantified using a colour histogram and calculated as a percentage of the total heart cross sectional area.

### Western blot analyses

All western blotting protocols were performed as previously described by us^78,80^. Since both DOX and SN are notorious producers of ROS^64,81^, we next investigated the effect of DOX and DOX + SN treatment on molecular markers of oxidative stress related damage, downstream anti-oxidant response targets and mitochondrial content and remodelling/biogenesis. Western blot experiments were performed from frozen SOL homogenates. SOL muscles were homogenized for 20-30 s in ice-cold Western Immunoprecipitation Kinase (WIK) buffer (40mMTris, pH 7.5; 1 mM EDTA; 5 mM EGTA; 0.5% TritonX-100; 25 mM β-glycerophosphate; 25 mM NaF; 1 mM Na_3_VO_4_; 10 μg/ml leupeptin; and 1 mM PMSF). Homogenate was centrifuged at 3,500 rpm for 5 min at 4 °C, before the pellet was resuspended and the whole muscle homogenate was used for further analysis. Protein concentrations were determined using a sample assay kit (Bio-Rad Laboratories, Hercules, CA, USA), to ensure equal loading on the gels. Samples were prepared with equivalent amounts of protein (20–40 μg) in Laemmli buffer, heated for 5 min at 95 °C, and subjected to electrophoretic separation on 7.5% or 12% SDS-acrylamide gels. The only exception to this was when probing for the Total OXPHOS cocktail antibody samples were heated for 5 min at 40 °C, as per supplier recommendations. Following electrophoretic separation, proteins were transferred to a polyvinylidene fluoride (PVDF) membrane, blocked with 5% not-fat milk powder in Tris-buffered saline containing 0.1% Tween 20 (TBST) for 1 h followed by an overnight incubation at 4 °C with primary antibody dissolved in TBST containing either 1% BSA or 3% non-fat milk powder. The following antibodies were used: anti-4-HNE (1:1,000; #ab46545, Abcam), Total OXPHOS cocktail (1:1,000; #ab110413, Abcam), anti-PGC-1β (1:500; #ab176328, Abcam), anti AMPK^Thr172^ (1:1,000, #2535, CST, anti-AMPK (1:1,000, #2603, CST), Cyt-c (1:2000; #11940, CST), anti-DJ-1 (1:1,000; #5933, CST), anti-Keap-1 (1:1,000; # 8047, CST), anti-NQO-1 (1:1,000; #62,262, CST), anti-NRF-2 (1:1,000; #12721, CST), anti-p62^Ser349^ (1:1,000; #95697, CST), anti-p62 (1:1,000; #5114, CST), anti-HO-1 (1:1,000; #ADI-SPA-894, Enzo Life sciences), anti-SOD-1 (1:2000; #ADI-SOD-101, Enzo Life Sciences) and anti-nitrotyrosine (1:1,000; #06–284, Millipore). After overnight incubation, the membranes were washed 3 separate times for 10 min each in TBST and then probed with a peroxidase-conjugated secondary antibody (1:5,000; anti-rabbit IgG, Vector Laboratories or 1:20,000; anti-mouse IgG, Vector Laboratories) for 1 h at room temperature. Following another set of 3 separate washes for 10 min in TBST, the blots were developed with a DARQ CCD camera mounted to a Fusion FX imaging system (Vilber Lourmat, Eberhardzell, Germany) using ECL Prime reagent (Amersham, Piscataway, NJ, USA). Once the images were captured, the membranes were stained with Coomassie Blue to verify equal loading of total protein in all lanes. Densitometric measurements were carried out using FusionCAPTAdvance software (Vilber Lourmat).

### Citrate synthase activity

CS activity was assessed as a marker of mitochondrial content on SOL homogenates prepared for WB analyses and as described by us previously^37,78^. Homogenates were added to reagent cocktail containing (100 mM TRIS buffer, 1 mM DTNB and 3 mM Acetyl CoA) and oxaloacetate (10 mM) was used to initiate the reaction in a plate-based spectrophotometer at 412 nm (25 °C for 5 m). CS activity was calculated using the extinction coefficient of 13.6^82^.

### Statistics

Data is presented as mean ± standard error of the mean, unless stated otherwise. Data sets were tested for normality using a Shapiro–Wilk test. For normal data, a one-way ANOVA with Tukey’s post-Hoc test was used to detect treatment differences for all data except for muscle and organ mass and cardiac muscle morphology data where a two-way ANOVA was used with treatment and muscle/organ type/cardiac morphology as factors. Where interactions were detected, one-way ANOVA was used for multiple comparisons. Repeated measures ANOVA’s were used to assess time-dependent effects on body weight and body composition and voluntary activity/calorimetry changes between the PRE and POST periods. An α-value of 0.05 was considered significant. Data was analysed using Graphpad prism (GraphPad Software, San Diego, CA 92,108, USA).

### Ethical approval

All experimental procedures were approved by the Victoria University Animal Ethics Committee (AEETH15/006) and conformed to the Australian Code of Practice for the Care and Use of Animals for Scientific Purposes.

## Supplementary information

Supplementary file1

Supplementary file2

Supplementary file3
