# Supplementary material for: Sodium nitrate co-supplementation does not exacerbate low dose metronomic doxorubicin-induced cachexia in healthy mice
Source: Sci Rep. 2020 Sep 24;10:15044. doi: 10.1038/s41598-020-71974-z (PMC7518269; doi:10.1038/s41598-020-71974-z)
Supplement: Supplementary file 1 — Supplementary file1 [file 41598_2020_71974_MOESM1_ESM.docx]

**Article Type**

Original Research Article

**Title**

Sodium nitrate co-supplementation does not exacerbate low dose metronomic doxorubicin-induced cachexia in healthy mice.

**Authors**

Dean G Campelj^12^, Danielle A Debruin^12^, Cara A. Timpani^12^, Alan Hayes^123^, Craig A. Goodman^124^, & Emma Rybalka^12^

^1^Institute for Health and Sport, Victoria University, Melbourne, Victoria, Australia

^2^Australian Institute for Musculoskeletal Science (AIMSS), Victoria University, St Albans, Victoria, Australia

^3^Department of Medicine - Western Health, Melbourne Medical School, The University of Melbourne, Melbourne, Victoria, Australia

^4^Centre for Muscle Research (CMR), Department of Physiology, The University of Melbourne, Parkville, Victoria, Australia

**Corresponding Author**

Dr Emma Rybalka

+ 61 3 83958226

emma.rybalka@vu.edu.au

**Keywords**

Chemotherapy, cachexia, sodium nitrate, doxorubicin


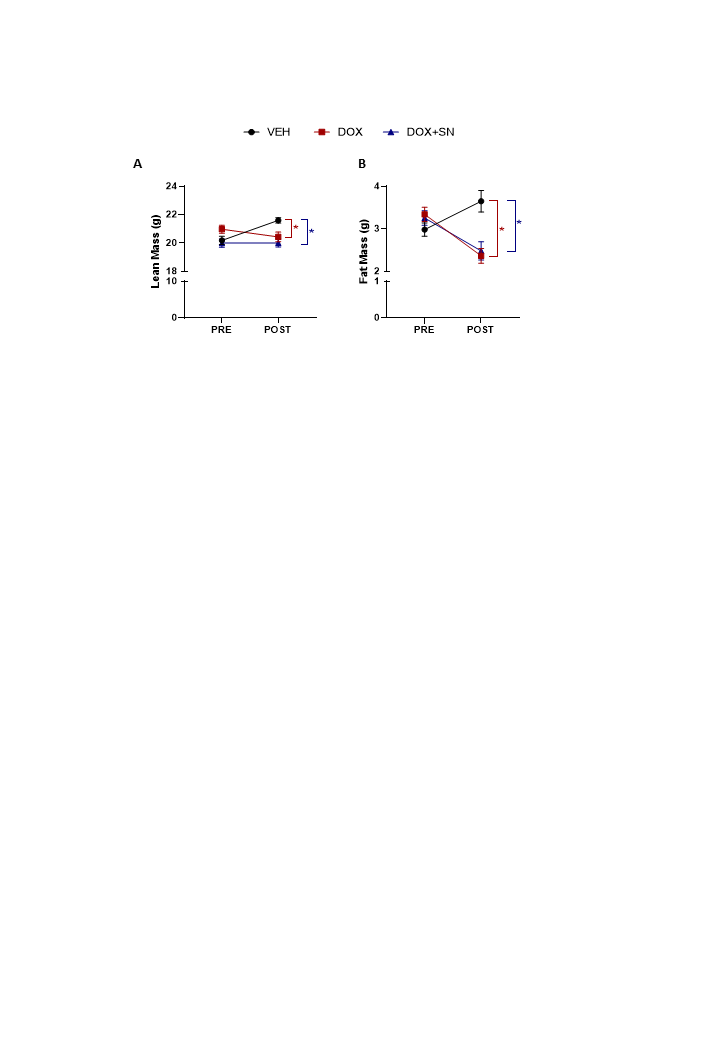


**Fig.S1 *The effect of LDM DOX administration and SN co-supplementation* *on lean and fat mass pre- and post-treatment*.** Body composition indices of **(A)** lean and **(B)** fat mass were measured pre and post treatment and were significantly reduced from DOX and DOX+SN treatment compared to VEH (**p*<0.05). *n*=7-8 per group.


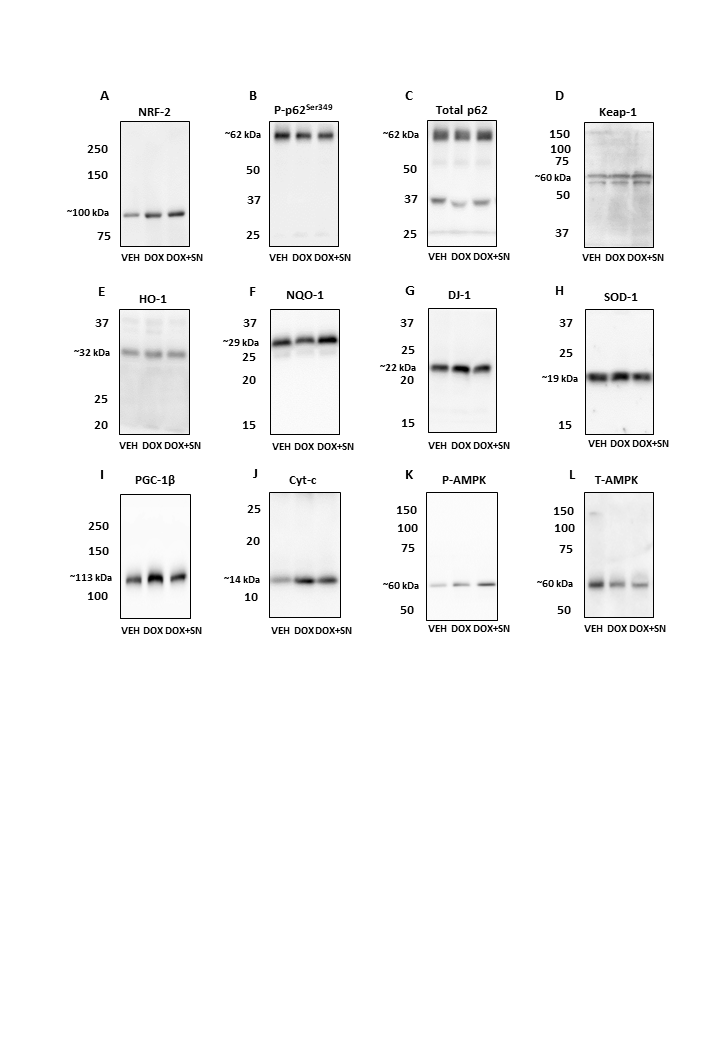


**Fig. S2 Full-length Western blot images relating to the data presented in Fig. 5 and 6.** After transferring the protein from the gel to the PVDF membrane, membranes were cut horizontally to allow for probing with multiple primary antibodies on a single membrane/gel. Subsequently, the above images display the largest available vertical membrane area probed with each respective primary antibody. The 4-HNE, nitrotyrosine and OXPHOS cocktail antibodies were not included here as their representative images already illustrate full length western blot images.
