# Supplementary figures and images for: Sodium nitrate co-supplementation does not exacerbate low dose metronomic doxorubicin-induced cachexia in healthy mice
Source: Sci Rep. 2020 Sep 24;10:15044. doi: 10.1038/s41598-020-71974-z (PMC7518269; doi:10.1038/s41598-020-71974-z)

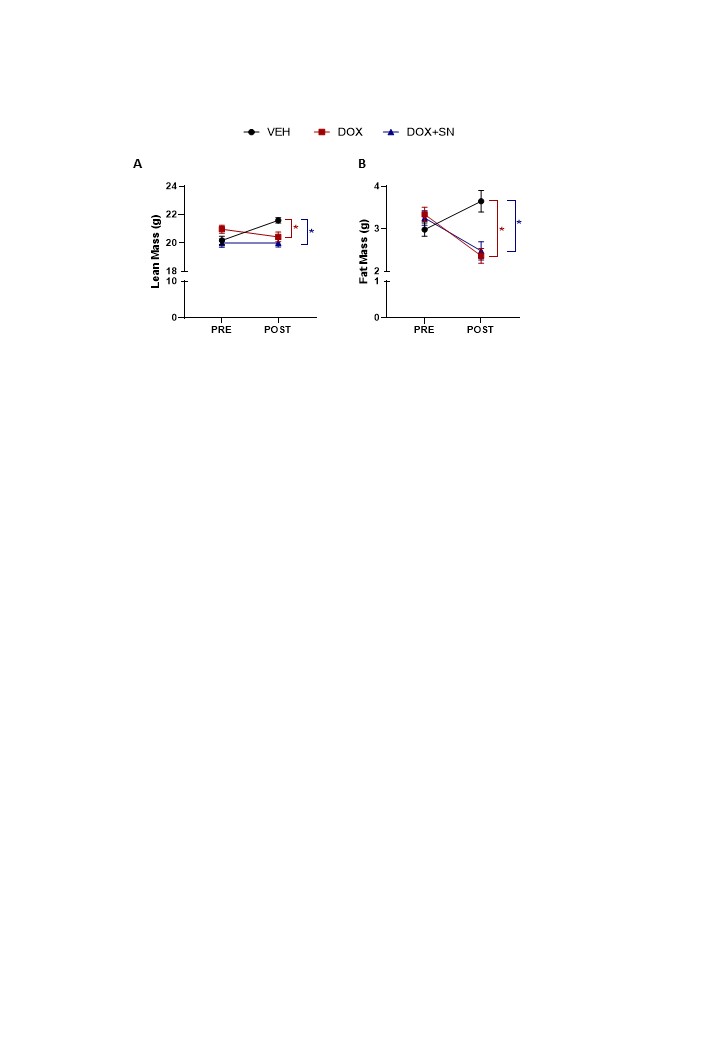

Supplement: Supplementary file 2 — Supplementary file2 [file 41598_2020_71974_MOESM2_ESM.jpg]

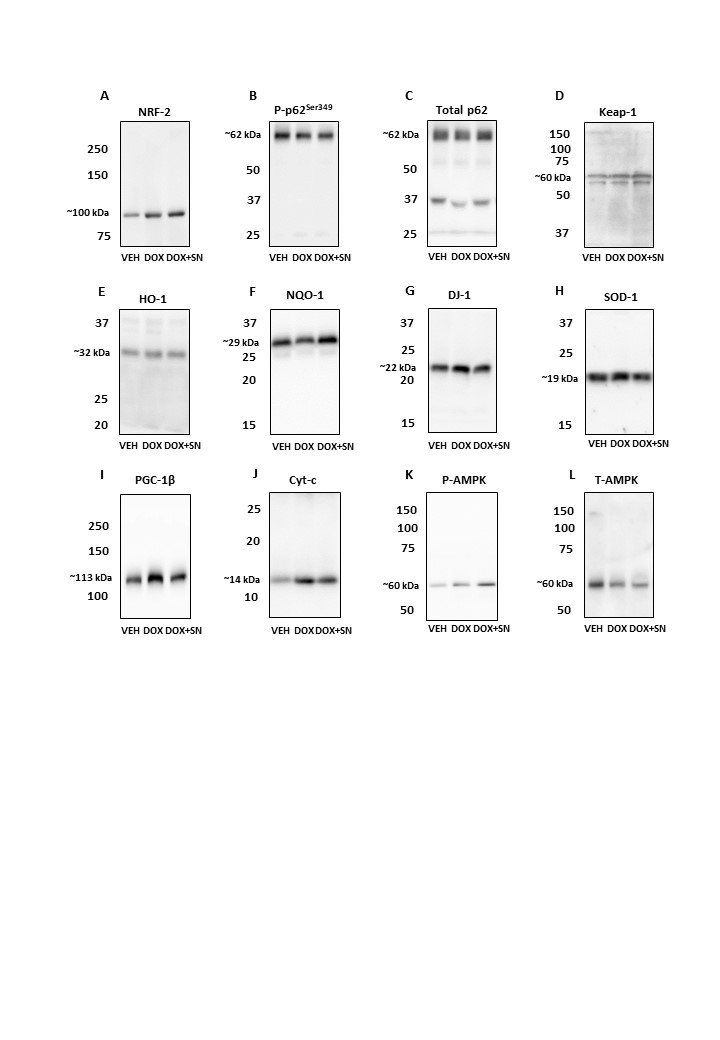

Supplement: Supplementary file 3 — Supplementary file3 [file 41598_2020_71974_MOESM3_ESM.jpg]
